# Simultaneous measurement of intra-epidermal electric detection thresholds and evoked potentials for observation of nociceptive processing following sleep deprivation

**DOI:** 10.1007/s00221-021-06284-5

**Published:** 2022-01-07

**Authors:** Boudewijn van den Berg, Hemme J. Hijma, Ingrid Koopmans, Robert J. Doll, Rob G. J. A. Zuiker, Geert Jan Groeneveld, Jan R. Buitenweg

**Affiliations:** 1 Biomedical Signals and Systems, Technical Medical Centre, University of Twente, Drienerlolaan 5, PO Box 217, 7500 AE Enschede, The Netherlands; 2 Centre for Human Drug Research, Zernikedreef 8, 2333 CL Leiden, The Netherlands; 3 Clinical Neuropharmacology, Leiden University Medical Center, Albinusdreef 2, 2333 ZA Leiden, The Netherlands

**Keywords:** Sleep deprivation, Sex, Pain, Nociceptive processing, Intra-epidermal electric stimulation, Detection threshold, Detection slope, Evoked potential, Linear mixed regression

## Abstract

Sleep deprivation has been shown to increase pain intensity and decrease pain thresholds in healthy subjects. In chronic pain patients, sleep impairment often worsens the perceived pain intensity. This increased pain perception is the result of altered nociceptive processing. We recently developed a method to quantify and monitor altered nociceptive processing by simultaneous tracking of psychophysical detection thresholds and recording of evoked cortical potentials during intra-epidermal electric stimulation. In this study, we assessed the sensitivity of nociceptive detection thresholds and evoked potentials to altered nociceptive processing after sleep deprivation in an exploratory study with 24 healthy male and 24 healthy female subjects. In each subject, we tracked nociceptive detection thresholds and recorded central evoked potentials in response to 180 single- and 180 double-pulse intra-epidermal electric stimuli. Results showed that the detection thresholds for single- and double-pulse stimuli and the average central evoked potential for single-pulse stimuli were significantly decreased after sleep deprivation. When analyzed separated by sex, these effects were only significant in the male population. Multivariate analysis showed that the decrease of central evoked potential was associated with a decrease of task-related evoked activity. Measurement repetition led to a decrease of the detection threshold to double-pulse stimuli in the mixed and the female population, but did not significantly affect any other outcome measures. These results suggest that simultaneous tracking of psychophysical detection thresholds and evoked potentials is a useful method to observe altered nociceptive processing after sleep deprivation, but is also sensitive to sex differences and measurement repetition.

## Introduction

Despite ample research efforts, there are only few biomarkers that can be used for objective monitoring and stratification of chronic pain patients. Patients with chronic pain often experience sensations of pain in response to a non-nociceptive input (i.e., allodynia), or an increased sensation of pain in response to a nociceptive input (i.e., hyperalgesia). A current challenge is to find biomarkers that can identify alterations in nociceptive processing leading to or involved in chronic pain on an individual level. The identification of such biomarkers could allow for patient stratification into functionally distinct groups, and may enable prediction of treatment efficacy per individual (Mouraux and Iannetti 2018). Furthermore, the development of such mechanism-based biomarkers can make it possible to accurately quantify the effects of analgesic drugs on nociceptive processing, which may provide an important proof-of-concept tool in early phase clinical pharmacology studies.

Key aspects in many types of chronic pain, including fibromyalgia, headache, and complex regional pain syndrome, are central sensitization (Woolf 2011) and reduced endogenous modulation of nociceptive input (Edwards 2005). Therefore, recent studies have focused on measuring the effect of central sensitization or reduced inhibition induced by experimental pain models, e.g., capsaicin-induced secondary hyperalgesia (Lee et al. 2008; Zambreanu et al. 2005). One method to centrally alter pain perception is by depriving healthy individuals of sleep (Schuh-Hofer et al. 2013). In this model, both central sensitization and reduced endogenous inhibition are thought to increase pain perception (Herrero Babiloni et al. 2020). Various studies have demonstrated a close relation between sleep impairments and an increased sensitivity to pain stimuli. In healthy subjects, sleep deprivation has been shown to cause hyperalgesic responses and an altered evoked cortical response, i.e., a decreased amplitude and increased habituation of the P2 in laser evoked potentials (Schuh-Hofer et al. 2013, 2015). Another recent study demonstrated impaired conditioned pain modulation and facilitation of temporal pain summation following 24 h of total sleep deprivation in healthy subjects (Staffe et al. 2019). Impaired pain inhibition on one hand, and enhanced pain facilitation on the other, have both been related to various chronic pain conditions such as musculoskeletal, visceral, and neuropathic pain (Herrero Babiloni et al. 2020). These observations suggest that sleep deficiency leads to altered central nociceptive processing, and an associated increase in pain perception. The sleep deprivation model may therefore be ideal to generate biomarkers that aim to quantify altered central nociceptive processing in healthy volunteer and chronic pain patient populations.

Recently, we developed a method for the characterization of both peripheral and central nociceptive processing by measuring the effect of nociceptive stimulus properties on detection probability and cortical evoked potentials (EPs). Nociceptive nerve fibers in the skin are activated using low-intensity intra-epidermal electric stimulation with cathodic square-wave pulses (Mouraux et al. 2010). Inhibition and facilitation of repeated nociceptive input are explored by varying the number of pulses and the inter-pulse interval (Doll et al. 2016a; Mouraux et al. 2014; van der Heide et al. 2009), based on the concept that central [e.g., temporal summation, short-term synaptic plasticity (Zucker and Regehr 2002)] or peripheral [e.g., subthreshold or suprathreshold super-excitability (Bostock et al. 2005)] neural mechanisms can attenuate or amplify neural activation by a second pulse dependent on its time with respect to the first pulse.

During a single measurement session, single- and double-pulse stimuli are applied according to an adaptive method of limits to track corresponding nociceptive detection thresholds (Doll et al. 2016a) while recording the electroencephalogram (EEG) to measure associated EPs (van den Berg et al. 2020). This combination of outcome measures potentially provides a unique insight into nociceptive processing. Nociceptive detection thresholds can be used to observe altered sensitivity (Doll et al. 2016b; Gottrup et al. 1998; Treede et al. 1992). In addition, the reliability of detecting the corresponding stimulus level (i.e., the minimum needed for a subject to detect nociception) is quantified by the detection probability slope (Gold and Ding 2013). EPs can be used as biomarker for altered nociception, such as in the case of central sensitization (van den Broeke et al. 2015), attentional modulation (Legrain et al. 2002), and placebo analgesia (Wager et al. 2006). We believe that both outcomes (i.e., EPs and nociceptive detection thresholds) measure different aspects of nociceptive processing and should be combined in a single experiment. After an initial demonstration that both techniques could be efficiently combined (van den Berg et al. 2020), we showed how the combined method may be used for studying the effect of intra-epidermal stimulus properties on nociceptive detection thresholds and EPs in a healthy population (van den Berg and Buitenweg 2021).

This combined method was developed with the goal of identifying combinations of psychophysical and neurophysiological features that could aid diagnosis and stratification of chronic pain patients, and as a proof-of-concept tool to characterize the effects of (investigational) analgesics in early phase clinical studies. Here, we examined if we could register altered nociceptive processing following sleep deprivation using this method in an exploratory study with 24 healthy male and 24 healthy female subjects. We study the feasibility of using the combination of nociceptive detection thresholds and EPs to observe altered nociceptive processing following sleep deprivation in both sexes.

## Methods

**Fig. 1:**
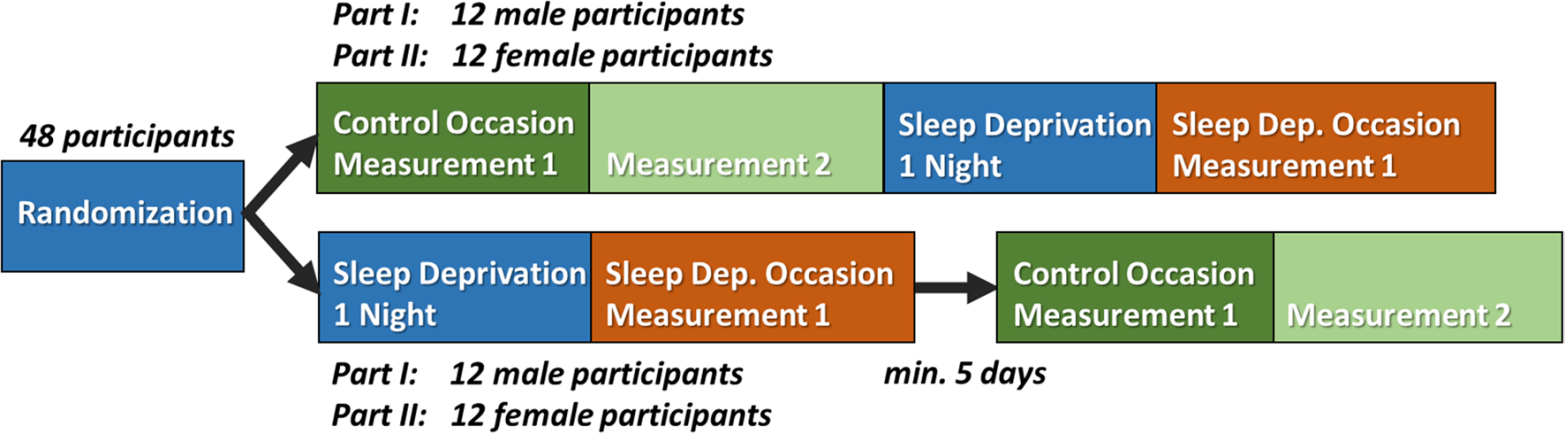
Participants were measured on two occasions: after a night of sleep deprivation (1 measurement) and after a normal night of sleep (2 measurements). If the sleep deprived occasion preceded the control occasion, a resting period of at least 5 days was used between both occasions

The work presented here was part of a study at the Centre for Human Drug Research (Leiden, The Netherlands) in which also other nociceptive pain tasks were performed. During the first part of this study, 24 male subjects were included. During the second part, 24 female subjects were included. In each part subjects participated in a measurement session (described below) after a night of sleep deprivation (sleep deprived occasion) and after a normal night of sleep (control occasion) (Fig. 1). On the sleep deprived occasion, subjects were deprived of their sleep by remaining awake a full night under supervision of a research assistant, after which the subjects participated in one measurement session in the morning. To ensure wakefulness of the subjects, they were closely monitored the entire night. To minimize the chance of creating a bias in study results, the interactions between subject and research assistant were kept to a minimum at night. In addition, the morning measurements were performed by a different assistant than the assistant that monitored the subject(s) during the sleep deprivation night. On the control occasion, subjects participated to two measurement sessions following a normal night of sleep, one in the morning and one in the afternoon. Participants were asked to go to sleep between 22:00 and 23:00, and to wake up between 7:00 and 8:00, on the night preceding the control occasion. The order of both occasions was randomized. If the sleep deprived occasion preceded the control occasion, a minimum resting period of at least 5 days was required. In practice, this resting period was either 7 or 8 days on all occasions.

The study received approval from a Medical Review and Ethics Committee (Foundation BEBO, Assen, The Netherlands) before study start, and was performed in accordance with the Declaration of Helsinki. All subjects provided written informed consent prior to any study assessments taking place. The study has prospectively been registered in the Dutch Trial Register (NTR) as NTR7517.

### Participants

A total of 24 healthy male (age 26.2 ± 2.1) and 24 healthy female (age 25.9 ± 3.0) participants were enrolled. Participants were recruited via media advertisement or from the subjects’ database of the Centre for Human Drug Research, Leiden, The Netherlands. Inclusion criteria were an age between 23 and 35 years, to reduce the potential influence of age on outcome measures, and a body mass index between 18 and 32 kg/m^2^, to exclude underweight or extremely overweight individuals. Exclusion criteria were a history or symptoms of any significant disease, history or presence of sleep disorders, a change in time zones 7 days prior to the study period, average usage of tobacco products equivalent to or more than 10 cigarettes per day, average usage of (methyl)-xanthines of more than 8 units per day, and inability to refrain from usage during the study occasions. No usage of (illicit) drugs was permitted from 3 days prior to each study period until discharge. Consumption of alcohol or tobacco- and nicotine-containing products was not permitted from 24 h prior to each scheduled visit until discharge. Participants underwent a urine drug screening and alcohol breath test on each arrival at the clinical research unit, i.e., before the start of each occasion. In addition, participants were not allowed to consume excessive amounts of caffeine, defined as more than 800 mg per day, from 2 days prior to each visit. Participants fully abstained from using caffeine-containing products from 4 h prior to each visit until discharge. No prescription medications and over-the-counter medications, except for contraceptive pill usage, were permitted within 14 days prior to the first occasion, or less than 5 half-lives, and during the course of the study. In addition, no vitamin, mineral, herbal, and dietary supplements were permitted within 7 days prior to the first occasion, or less than 5 half-lives, and during the course of the study.

To minimize a possible influence of the menstrual cycle on pain perception, females were required to use a reliable method of hormonal contraception at least 30 days before the first study day until the end of the study. Females were required to use their own hormonal anticonception (prescribed by their general practitioner of gynecologist) continuously during study participation or were only allowed to participate if the study days were more than 2 days after re-start of contraceptive pill use or after bleeding withdrawal. This to prevent possible variations caused by the menstrual cycle. No side effects of hormonal contraception were reported.

### Stimuli

**Fig. 2:**
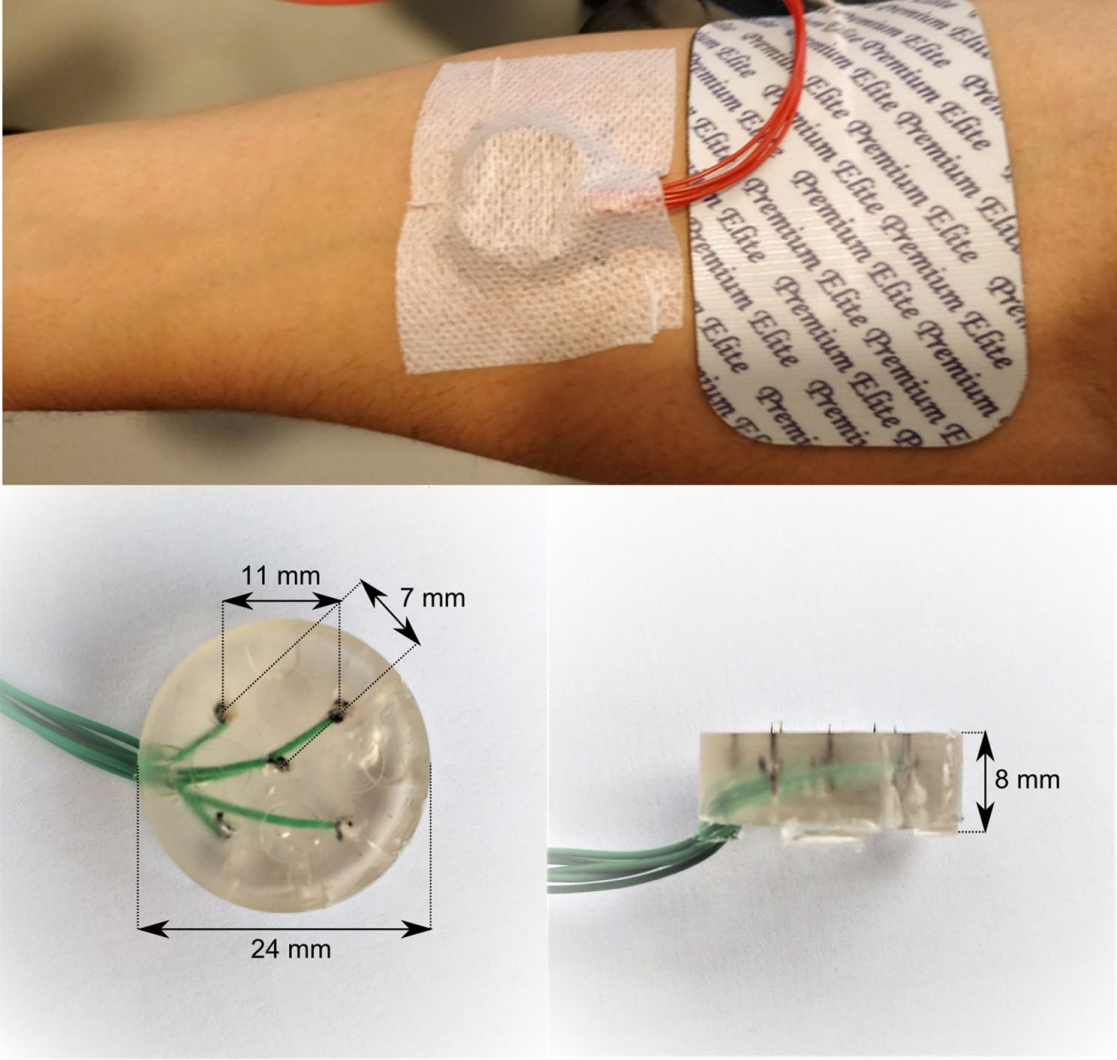
Electrode placement on the volar forearm on the side of the dominant hand (top) and electrode dimensions (bottom)

Participants received intra-epidermal electric pulses applied by a constant current stimulator (NociTRACK AmbuStim, University of Twente, Enschede, The Netherlands). Intra-epidermal electric stimulation at intensities of less than twice the detection threshold preferentially activates Aδ-fibers in the skin (Motogi et al. 2016; Mouraux et al. 2010; Poulsen et al. 2020). Stimuli were applied via an electrode attached to the volar lower arm at the side of the dominant hand (Fig. 2). The electrode consisted of an array of 5 interconnected microneedles embedded in silicone, each needle protruding 0.5 mm from the electrode surface. Previous studies using this electrode showed that stimulation resulted in a sharp pricking sensation (Steenbergen 2012), and similar latencies of response times and evoked N1, N2 and P2 peaks in comparison with earlier studies using intra-epidermal and laser stimulation (van den Berg and Buitenweg 2021). In addition to single-pulse stimuli, double-pulse stimuli were used to observe potential effects of inhibition or facilitation of repeated nociceptive input (Doll et al. 2016a; Mouraux et al. 2014; van der Heide et al. 2009). As such, two stimulus types were used in this study:

- A single 210 µs pulse
- A double 210 µs pulse with an inter-pulse interval (IPI) of 10 ms.

### Procedure

While seated in a comfortable chair, participants were instructed to focus their attention on the stimulation electrode, to reduce the potential influence of (variations in) spatial attention. First, a rough estimate of the detection threshold was obtained using a normal staircase procedure with a stepsize of 0.025 mA. The participant was instructed to hold a button, and to release the button as soon as a stimulus was perceived. Second, an accurate estimate of the detection threshold was obtained using an adaptive and randomized psychophysical method of limits, also referred to as ‘threshold tracking’, designed to estimate detection thresholds with a potential drift (Doll et al. 2015). Participants were instructed to hold a button, and to briefly release the button when a stimulus was perceived. A vector of 5 stimulus amplitudes was initialized with a stepsize of 0.025 mA around the initial estimate of the detection threshold. For each stimulus, a value was randomly chosen from this vector. When the stimulus was detected, the vector was decreased by 0.025 mA. When the stimulus was not detected, the vector was increased by 0.025 mA. This process was repeated for a total of 180 single- and 180 double-pulse stimuli, during a time period of approximately 20 min. The interval between two consecutive stimuli was randomized with a uniform distribution of 2.5–3.5 s.

### Electroencephalography recording

During the entire detection threshold tracking procedure, the scalp EEG was recorded at 32 Ag/AgCl electrodes located on the scalp according to the international 10/20 system. Electrode impedance was kept below 5 kΩ. To reduce eye blink and movement artefacts, participants were asked to fix their gaze at one spot on the wall and blink as few times as possible while pressing the response button and focusing their attention on the received stimuli.

### Analysis

#### Effect of stimulus properties and sleep deprivation on detection probability

The effect of stimulus properties and sleep deprivation on the detection probability was analyzed for the male group, female group, and the combination of both groups using a generalized linear mixed model in R, estimated using the lme4 (Bates et al. 2015) and MASS toolboxes (Venables and Ripley 2002). We used the statistical model in (1), where the log-odds of stimulus detection ($\mathrm{ln}\left(\frac{{\mathrm{P}}_{\mathrm{d}}}{1-{\mathrm{P}}_{\mathrm{d}}}\right)$) is modulated by the effects and interaction of stimulus type ($\mathrm{TYP})$, i.e., single- or double-pulse, stimulus amplitude ($\mathrm{AMP}$) and condition ($\mathrm{C}$) and by the effects and interaction of trial number ($\mathrm{TRL}$) and condition ($\mathrm{C}$). We also added terms for measurement number (M) and occasion (O) to account for potential confounding. Condition, measurement, and occasion were modeled as categorical. All within-subject fixed effects were also included as random effects grouped by subject ($\mathrm{S}$) to effectively account for differences between subjects (Barr et al. 2013)

1 $$\ln \left( {\frac{{P_{d} }}{{1 - P_{d} }}} \right) \sim 1 + AMP * TYP * C + TRL * C + M + O + (1 + AMP * TYP * C + TRL * C + M + O|S).$$

Before GLMR analysis of the dataset, outliers were excluded, defined as measurements in which the detection threshold was estimated smaller than 0 or larger than 1.6 mA, or where the slope of the psychometric curve was estimated smaller than 0 or larger than 100 mA^−1^. Effect significance was tested using a two-tailed type-III test using Wald–Chi-square statistics.

Detection thresholds and slopes were computed using the estimated model coefficients. Differences of detection thresholds and slopes between the sleep deprived measurement and the first control measurement and between both control measurements were tested by generating a posterior distribution of each model coefficient with 20,000 samples using the ARM package in R (Gelman and Hill 2006). Subsequently, these posterior distributions were used to compute the distribution, confidence intervals, and significance of the (difference between) detection thresholds.

#### Preprocessing of EEG data

The scalp EEG data was pre-processed using Fieldtrip (Oostenveld et al. 2011). Epochs were extracted from the EEG from 0.5 s before to 1.0 s after the stimulus. Eye blink and movement artefacts were identified and removed using independent component analysis (Delorme et al. 2007), resulting in removal of 2 independent components on average. Epochs with excessive EMG activity were excluded from analysis based on visual inspection. Subsequently, epochs were bandpass-filtered from 0.1 to 40 Hz and baseline-corrected using the interval ranging from − 0.5 s to 0.0 s relative to stimulus onset.

#### Grand average evoked potential

The Cz-M1M2 derivation was used for analysis of the central EP, as previous studies showed that these channels (Cz, M1, and M2) have the largest SNR for intra-epidermal electric EPs in healthy participants, when using a 32-channel electrode configuration (van den Berg and Buitenweg 2021). Grand average waveforms at the identified latency at the Cz-M1M2 derivation were computed by averaging all trials seperated by measurement number (1 or 2), stimulus type (single- or double-pulse), and condition (with or without sleep deprivation), resulting in 180 trials per average. A positive peak (P2) was defined as the most positive peak between 300 and 500 ms at Cz-M1M2 and selected for further analysis. The differences of average EP at Cz-M1M2 between the sleep deprived measurement and the first control measurement and between both control measurements were tested at the identified P2 latency (390 ms) using a two-tailed paired-sample *t* test.

#### Effect of stimulus properties on evoked potential

The effect of stimulus properties and sleep deprivation on the EP at P2 latency was analyzed for the male group, female group, and the combination of both groups using a linear mixed model in Matlab (version 2017b, MathWorks, Inc.). We used the statistical model in (2), similar to the model for analysis of detection probability in (1), but including a term for additional cortical activity evoked by stimulus detection (D) which could decrease with respect to the trial number (TRL), and also vary with respect to condition (C). Condition, stimulus detection, measurement, and occasion were modeled as categorical

2 $$U_{EEG} \sim 1 + AMP * TYP * C + TRL * D * C + M + O + (1 + AMP * TYP * C + TRL * D * C + M + O|S).$$

Significance of the effect coefficients was assessed using a two-tailed *t* test using Satterthwaite’s method for estimation of the degrees of freedom.

## Results

### Exclusion of outliers

In the first part of the study (males), 7 out of 72 measurements were excluded due to an incomplete measurement, as a result of technical problems with the measurement setup. For the analysis of EEG, 3 out of the remaining 65 measurements were excluded due to extreme noise caused by a faulty electrode. For the analysis of detection probability, 16 out of the remaining 65 measurements were excluded due to poor task performance leading to unreliable detection thresholds or slopes as defined in the section “Effect of stimulus properties and sleep deprivation on detection probability”.

In the second part of the study (females), 4 out of 72 measurements were excluded due to an incomplete measurement, as a result of technical problems with the measurement setup. For the analysis of EEG, 3 out of the remaining 68 measurements were excluded due to extreme noise caused by a faulty electrode. For the analysis of detection probability, 2 out of the remaining 68 measurements were excluded due to poor task performance leading to unreliable detection thresholds or slopes as defined in the section “Effect of stimulus properties and sleep deprivation on detection probability”.

### Effect of stimulus properties and sleep deprivation on detection probability

The effect of stimulus properties and sleep deprivation on detection probability is shown in Table 1. The random-effects covariance matrices associated with each generalized linear mixed model fit are available in Appendix I. In all groups, significant effects on the detection probability were observed for the intercept, amplitude, type, trial number, and the interaction between amplitude and type. The detection probability increases with respect to the amplitude and decreases over the number of trials. The positive coefficients for type and the interaction between amplitude and type shows that addition of a second pulse to the stimulus increases detection probability. An additional significant effect of stimulus type is observed in the combined group, as well as male group only. The combination of both groups and the female group show an additional significant effect of measurement, and of the interaction between amplitude, type, and condition.

**Table 1:** Effect of stimulus properties on the detection probability for the male group (M), the female group (F), and the combination of both (All), computed using GLMR

Stimulus property	Coeff. (All)	Coeff (M)	Coeff (F)	χ^2^ (All)	χ^2^ (M)	χ^2^ (F)	*p* (All)	*p* (M)	*p* (F)
(Intercept)	**− 3.50**	**− 3.19**	**− 3.44**	**172.51**	**80.24**	**69.25**	** < 0.001**	** < 0.001**	** < 0.001**
Amplitude (AMP)	**6.10**	**4.45**	**7.52**	**148.42**	**85.06**	**98.05**	** < 0.001**	** < 0.001**	** < 0.001**
Type (TYP)				**6.01**	**11.83**	0.66	** < 0.05**	** < 0.001**	0.42
Type 2	**− 0.39**	**− 0.85**	**− **0.19						
Trial number (TRL)	**− 0.52**	**− 0.41**	**− 0.62**	**108.89**	**26.88**	**104.33**	** < 0.001**	** < 0.001**	** < 0.001**
Measurement (M)				**5.90**	0.82	**4.33**	** < 0.05**	0.37	** < 0.05**
Measurement 2	**0.64**	0.31	**0.77**						
Occasion (O)				2.22	0.97	2.10	0.14	0.32	0.14
Occasion 2	0.30	**− **0.48	**− **0.32						
Condition (C)				0.11	1.64	0.01	0.74	0.20	0.90
Sleep Dep	0.14	0.85	0.08						
Amplitude × Type				**52.81**	**20.69**	**38.09**	** < 0.001**	** < 0.001**	** < 0.001**
Amplitude × Type 2	**6.69**	**5.74**	**7.81**						
Amplitude × Condition				1.09	0.82	1.07	0.30	0.36	0.30
Amplitude × Sleep Dep	1.23	1.14	1.88						
Type × Condition				0.10	1.40	0.70	0.75	0.23	0.40
Type 2 × Sleep Dep	**− **0.13	0.54	**− **0.60						
Trial number × Condition				0.06	0.06	0.16	0.80	0.81	0.69
Trial number × Sleep Dep	**− **0.02	**− **0.03	**− **0.04						
Amplitude × Type × Condition				**3.74**	0.52	**3.90**	**0.05**	0.47	** < 0.05**
Amplitude × Type 2 × Sleep Dep	**3.18**	1.36	**5.19**						

Significance was assessed using type-III Wald–Chi-square statistics with one degree of freedom

All effect coefficients are expressed in log-odds per unit with the units ${\mathrm{mA}}^{-1}$ for amplitude and ${\left(100\mathrm{ trials}\right)}^{-1}$ for trial number

The numbers of measurement and occasion refer to the moments at which the procedure was conducted as described in Fig. 1. Significant values (*p* < 0.05) are shown in bold.

Detection thresholds derived from the coefficient estimates are shown in Table 2. For the combined group and the male group, the estimate of the detection threshold is significantly lower for both single-pulse and double-pulse stimuli after sleep deprivation. The female group shows a similar non-significant trend after sleep deprivation. For the combination of both groups and the female group, the estimate of the detection threshold is significantly lower for both single-pulse and double-pulse stimuli during the second control measurement. The male group shows a similar non-significant trend during the second control measurement.

**Table 2:** Detection thresholds for the male group (M), the female group (F), and the combination of both (All) per stimulus type (in $\mathrm{mA}$)

Stimulus type	Thresh (All)	Thresh (M)	Thresh (F)	95% CI (All)	95% CI (M)	95% CI (F)
Single-pulse, Control 1	0.57	0.72	0.46	[0.48 0.69]	[0.55 0.94]	[0.37 0.55]
Single-pulse, Control 2	0.47*	0.65	0.35*	[0.38 0.57]	[0.43 0.92]	**[0.31 0.41]**
Single-pulse, Sleep Dep	0.46*	0.42**	0.36	**[0.38 0.58]**	**[0.28 0.62]**	[0.29 0.48]
Double-pulse, Control 1	0.30	0.40	0.24	[0.25 0.38]	[0.29 0.59]	[0.19 0.29]
Double-pulse, Control 2	0.25*	0.37	0.19*	[0.21 0.32]	[0.24 0.58]	**[0.16 0.22]**
Double-pulse, Sleep Dep	0.23*	0.21***	0.18	**[0.18 0.29]**	**[0.14 0.31]**	[0.14 0.24]

Control 1 and Control 2 refer to the first and second control measurement in Fig. 1, respectively

Each significant difference of the sleep deprived measurement or the second control measurement with respect to the first control measurement is denoted with *(*p* < 0.05), **(*p* < 0.01), and ***(*p* < 0.001)

Detection thresholds with a significant difference with respect to the first control occasion (*p* < 0.05) and associated confidence intervals are shown in bold.

Detection probability slopes derived from the coefficient estimates are shown in Table 3. The slope appears to increase in all groups after sleep deprivation. However, this increase was only significant in the female group for double-pulse stimuli.

**Table 3:** Detection probability slopes for the male group (M), the female group (F), and the combination of both (All) per stimulus type (in ${\mathrm{mA}}^{-1}$)

Stimulus type	Slope (All)	Slope (M)	Slope (F)	95% CI (All)	95% CI (M)	95% CI (F)
Single-pulse, Control 1 & 2	6.11	4.45	7.52	[5.14 7.07]	[3.56 5.35]	[6.04 8.99]
Single-pulse, Sleep Dep	7.32	5.59	9.42	[5.23 9.49]	[3.26 7.95]	[6.34 12.44]
Double-pulse, Control 1 & 2	12.79	10.18	15.33	[10.46 15.13]	[7.06 13.30]	[12.34 18.33]
Double-pulse, Sleep Dep	17.18	12.66	22.40*	[12.43 22.00]	[7.75 17.68]	**[15.80 28.84]**

Control 1 and Control 2 refer to the first and second control measurement in Fig. 1, respectively. Each significant difference of the sleep deprived measurement with respect to the control measurements is denoted with *(*p* < 0.05), **(*p* < 0.01), and ***(*p* < 0.001). Slopes with a significant difference with respect to both control occasions (*p* < 0.05) and associated confidence intervals are shown in bold.

### Grand average evoked potential

**Fig. 3:**
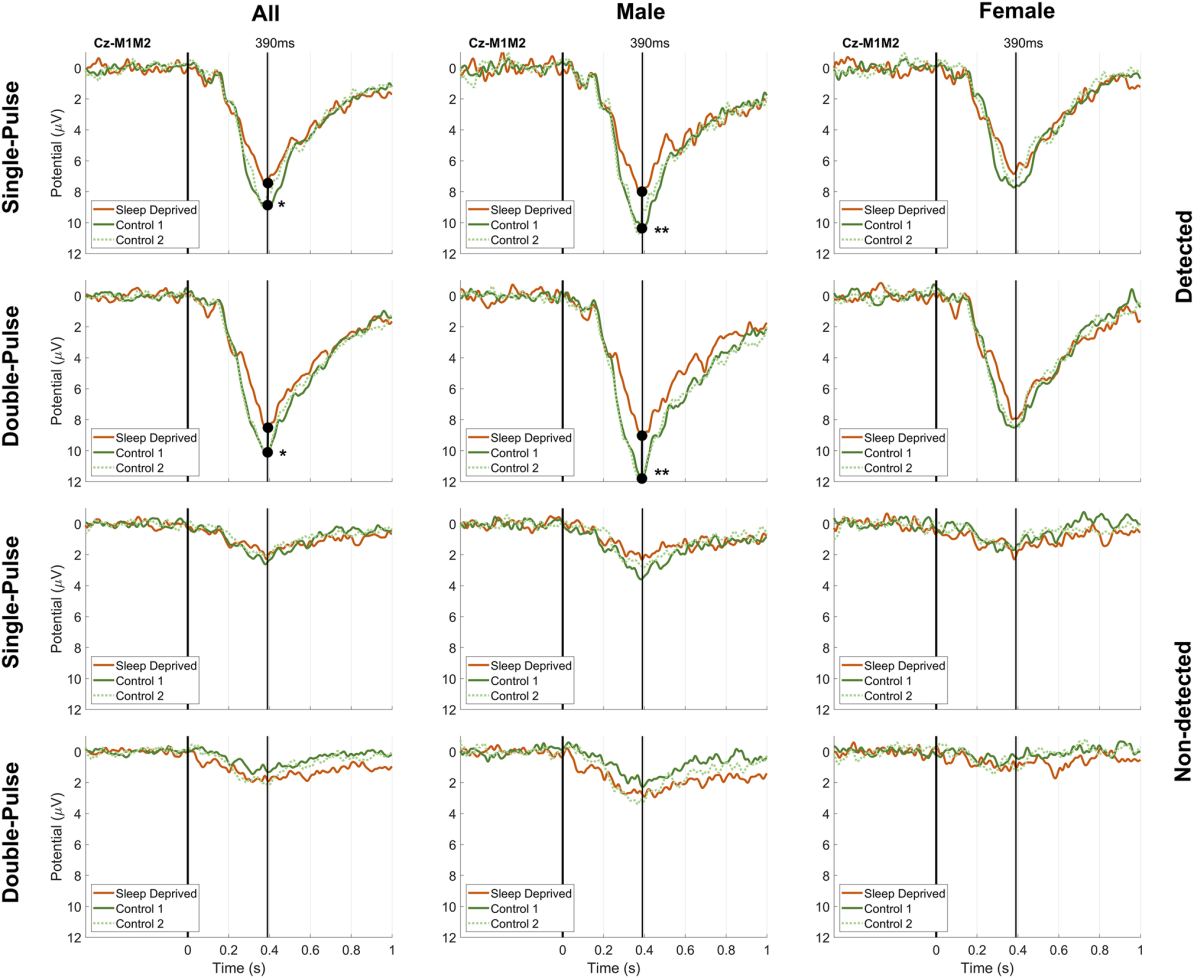
Grand average EP in each group in response to single-pulse and double-pulse intra-epidermal stimuli at Cz-M1M2 for participants with normal sleep during a first and a second measurement (Control M1 and Control M2, respectively) and after 24 h of sleep deprivation. There was a significant difference in maximum EP amplitude at Cz-M1M2 between the sleep deprived and the first control measurement for detected single- and double-pulse stimuli in the male group and the combination of both groups. Significance is indicated with *(*p* < 0.05) and **(*p* < 0.01)

The difference between sleep deprived and control measurements for each group is shown in the time domain at the Cz-M1M2 derivation in Fig. 3. For the combination of both groups and the male group, there was a significant decrease in maximum EP amplitude in response to detected single- and double-pulse stimuli after sleep deprivation. For the female group, there was no significant difference in maximum EP amplitude between sleep deprived on control measurements. For all groups, there was no significant difference in EP between both control measurements.

### Effect of stimulus properties and sleep deprivation on evoked potential

The effects of stimulus properties and sleep deprivation on the EP at 390 ms latency on the Cz-M1M2 derivation were quantified by linear mixed regression based on Eq. (2) and a *t* test of each computed effect coefficient. Results for each group are shown in Table 4. The random-effects covariance matrices associated with each linear mixed model fit are available in Appendix I. For each group, significant effects of stimulus properties on the EP were found for stimulus detection, trial number, amplitude, and the interaction between amplitude and type. For the combination of both groups and for the male group, a significant interaction between sleep deprivation and stimulus detection was found. For this interaction between sleep deprivation and stimulus detection, effect coefficients of − 1.28 and − 2.21 were found for the combination of both groups and for the male group, respectively, which means that the EP in response to detected stimuli decreased by − 1.28 and − 2.21 µV after sleep deprivation.

**Table 4:** The coefficient estimates, *t* values, and corresponding p values for the effect of stimulus properties on the EP at 390 ms (Cz-M1M2) in the male group (M), the female group (F), and the combination of both (All)

Stimulus property	Coeff (All)	Coeff (M)	Coeff (F)	*t* (All)	*df*	*T* (M)	*df*	*t* (F)	*df*	*p* (All)	*p* (M)	*p* (F)
(Intercept)	0.89	1.61	0.06	1.54	*31.2*	1.49	*15.6*	0.12	*50.8*	0.13	0.15	0.91
Detection (D)												
Detected	**7.02**	**7.71**	**6.43**	**11.10**	**44.6**	**7.45**	**22.0**	**8.45**	**21.7**	** < 0.001**	** < 0.001**	** < 0.001**
Amplitude	**2.40**	**2.54**	**2.58**	**4.11**	**35.1**	**2.60**	13.5	**3.04**	**34.0**	** < 0.001**	** < 0.05**	** < 0.01**
Type												
Type 2	− 0.37	− 0.59	− 0.50	− 1.04	108.3	− 1.05	48.9	− 0.85	29.8	0.30	0.30	0.40
Trial number (TRL)	− **0.57**	− **0.54**	**-0.65**	− **3.34**	**53.3**	**− 2.30**	**27.1**	− **2.62**	**25.8**	** < 0.01**	** < 0.05**	** < 0.05**
Measurement (M)												
Measurement 2	− 0.17	− 0.76	0.18	− 0.42	23.4	− 1.22	14.8	0.37	*18.5*	0.68	0.24	0.72
Occasion (O)												
Occasion 2	− 0.44	− 0.53	0.14	− 1.38	21.0	− 1.17	13.5	0.30	13.5	0.18	0.26	0.77
Condition (C)												
Sleep dep	− 0.42	− 1.57	0.03	− 0.46	29.8	− 1.05	16.1	0.02	19.1	0.65	0.31	0.98
Amplitude × Type												
Amplitude × Type 2	**3.60**	**3.60**	**4.65**	**4.08**	**11.3**	**4.22**	**12.1**	**2.37**	**11.7**	** < 0.01**	** < 0.01**	** < 0.05**
Trial number × Detection												
Trial number × Detected	− 0.55	− 0.34	− 0.72	− 1.96	43.7	− 0.84	21.1	− 1.85	23.4	0.06	0.41	0.08
Detection × Condition												
Detected × Sleep dep	− **1.28**	− **2.21**	− 0.99	− **2.39**	**45.3**	− **3.13**	**22.7**	− 1.28	23.3	** < 0.05**	** < 0.01**	0.21
Amplitude × Condition												
Amplitude × Sleep dep	0.56	1.61	0.61	0.58	25.4	1.00	14.0	0.37	11.7	0.57	0.34	0.72
Type × Condition												
Type 2 × Sleep dep	0.36	0.44	− 1.07	0.49	44.0	0.43	31.1	− 0.98	23.3	0.63	0.67	0.34
Trial number × Condition												
Trial number × Sleep dep	− 0.09	− 0.65	0.20	− 0.30	44.0	− 1.52	22.5	0.45	23.2	0.76	0.14	0.65
Amp. × Type × Condition												
Amp. × Type 2 × Sleep dep	− 1.29	− 0.33	5.24	− 0.97	16.8	0.25	13.5	1.54	16.4	0.35	0.81	0.14
Trial num. × Det. × Cond												
Trial num. × Det. × Sleep dep	− 0.45	− 0.14	− 0.69	− 0.93	45.9	− 0.21	26.9	− 0.98	21.8	0.36	0.83	0.34

All effect coefficients are expressed in $\mathrm{\mu V}$ per unit with the units ${\mathrm{mA}}^{-1}$ for amplitude and ${\left(100\mathrm{ trials}\right)}^{-1}$ for trial number

The numbers of measurement and occasion refer to the moments at which the procedure was conducted as described in Fig. 1

Significant values (*p* < 0.05) are shown in bold.

## Discussion

In search of a composite biomarker for altered nociceptive processing, we combined techniques to simultaneously measure detection thresholds and EPs in response to nociceptive intra-epidermal electric stimulation. We explored if this combination of techniques could be used to observe changes in nociceptive processing following sleep deprivation in a male and female population. We found that intra-epidermal electric detection thresholds and EPs both decreased after 24 h of sleep deprivation in a combined group of healthy male and female subjects.

The effects of intra-epidermal electric stimulus properties on the detection probability were similar to the effects observed in the previous studies (Doll et al. 2016a; van den Berg and Buitenweg 2021; van den Berg et al. 2020), supporting the validity of our results. Similar to these earlier observations on unchallenged healthy subjects, we observed a general positive effect of stimulus amplitude and the interaction between amplitude and type on detection probability (Table 1). Both effects indicate that the detection probability increased when the stimulus amplitude of single- or double-pulse stimuli increased, which is associated with an increased recruitment of peripheral nerve fibers at increased currents. The detection probability also increased following addition of a second pulse as a result of the temporal summation of neural activity elicited by both pulses, which was signified by the positive effect of stimulus type and the positive interaction between stimulus amplitude and stimulus type in generalized linear mixed regression (Table 1). The detection probability decreased over the number of trials, plausibly due to a decreased attention or physiological habituation to the stimulus. In addition, there was a significant interaction between stimulus amplitude, type, and sleep deprivation for the mixed population, suggesting that the effect of adding a second pulse on the detection probability is increased after sleep deprivation. This interaction suggests an increased facilitation or decreased inhibition of neural activity evoked by the second pulse following sleep deprivation. A potential explanation for increased facilitation of the second pulse is increased temporal summation, as originally defined by (Price et al. 1977), which has also been shown to be increased following sleep deprivation using modern temporal summation paradigms (Matre et al. 2016; Smith et al. 2019).

Nociceptive detection thresholds for intra-epidermal electric stimulation were decreased following sleep deprivation. These detection thresholds were computed from generalized linear mixed regression coefficients (Moscatelli et al. 2012), and statistically tested through Monte Carlo simulation of detection threshold distributions. As a result, we found that in a mixed population (i.e., male and female groups combined) detection thresholds for both types of stimuli decreased after sleep deprivation. Earlier studies have examined the effects of sleep deprivation using mechanical and thermal pain (detection) thresholds. Some of these studies support that pain thresholds are decreased following sleep deprivation, having observed a significant decrease in mechanical (Moldofsky and Scarisbrick 1976; Moldofsky et al. 1975; Onen et al. 2001) and heat pain thresholds (Kundermann et al. 2004) due to sleep deprivation. However, not all studies found a significant correlation between pain thresholds and sleep deprivation (Drewes et al. 1997; Older et al. 1998). We demonstrated here that the nociceptive intra-epidermal electric detection thresholds to single-pulse and double-pulse stimuli were decreased in a mixed population, while noting that both detection thresholds were also significantly decreased during the second control measurement. As such, any repeated measures designs involving nociceptive detection thresholds should account for this effect by randomization of the measurement order.

Intra-epidermal stimulation evoked a cortical response with a maximum at 390 ms, which was decreased following sleep deprivation. The latency of this evoked response was similar to the P2 potential measured in response to nociceptive stimuli in previous studies (Liang et al. 2016; Mouraux et al. 2014; van den Berg and Buitenweg 2021). We used the Cz-M1M2 derivation to study the influence of sleep deprivation and stimulus properties on evoked cortical activity at this latency. We found a significant decrease of the P2 amplitude in response to detected single- and double-pulse stimuli after sleep deprivation, while the waveform remained similar during both control measurements. Regression analysis showed a significant interaction between sleep deprivation and stimulus detection, suggesting that sleep deprivation mainly resulted in a reduction of task-related cortical activity.

A decrease of P2 amplitude at Cz-M1M2 has also been related to reduced stimulus intensity and reduced stimulus salience in earlier studies (Iannetti and Mouraux 2010; Ohara et al. 2004), which appears contradictory to the notion that sleep deprivation causes hyperalgesia (Lautenbacher et al. 2006). A decreased P2 amplitude might reflect a decreased attention (Legrain et al. 2003), as a result of sleep deprivation. However, decreased attention appears contradictory to our observation that sleep deprivation results in a higher nociceptive detection thresholds, which suggests that participants are more sensitive to nociceptive input following sleep deprivation. This simultaneous increase of sensitivity and decrease of measured cortical activity was also found in three recent studies assessing pain sensitivity (Azevedo et al. 2011; Ødegård et al. 2015; Schuh-Hofer et al. 2013). Hypotheses for this phenomenon in these studies include loss of attention or a reduction in cortical cognitive or perceptual mechanisms. However, a recent fMRI study suggests the reduction of cortical activity following sleep deprivation is associated with a reduction of stimulus evoked activity in the insula and the anterior cingulate cortex, which are both involved in the endogenous modulation of pain (Krause et al. 2019). Although the origin of this phenomenon is reason of debate, it shows that detection thresholds and EPs are measuring distinct aspects of nociceptive processing and are useful to combine to study effects of sleep deprivation on nociception. Further experimental and modelling studies are necessary to better explain why an increased nociceptive sensitivity and a decreased EP are both observed following sleep deprivation in this and other studies.

To the best of our knowledge, this is the first study to examine the effect of sleep deprivation on nociceptive detection thresholds and EPs in both a male and a female population. In fact, a few studies have been done to identify sex differences in nociceptive processing before and after sleep deprivation (Eichhorn et al. 2018; Smith et al. 2019). To start with, there was a large difference in detection task performance between males and females, as a total of 16 measurements had to be removed due to unreliable detection thresholds in the male group in comparison to only 2 measurements in the female group. This difference was also observed in the detection slopes [quantifying detection (un)certainty], which were lower for male subjects on all occasions. Furthermore, this difference between both groups was larger on the control occasion than on the sleep deprived occasion. The observed difference in task performance might be attributed to a greater sensitivity to noxious stimuli in females (Fillingim and Maixner 1995). However, other sex-related differences in sensitivity, cognitive performance, and attention cannot be excluded based on the current results.

Separate analysis of the results for a male and a female population suggests that outcomes are dependent on sex. While average detection thresholds decreased for both stimulus types in both groups, this decrease was only significant in the male population when analyzed in separate groups. On the other hand, only the female population showed an increased effect of double-pulse stimuli on detection probability following sleep deprivation, potentially associated with increased temporal summation of pulses. The grand average EP amplitude was significantly decreased after sleep deprivation in the male population and regression analysis showed a significant decrease in task-related activity following sleep deprivation in the male population only. Divergent sex-dependent effects of sleep deprivation on nociceptive processing and pain have been noted previously. Smith et al. (2019) observed that a significant increase of capsaicin-induced secondary hyperalgesia following sleep deprivation only occurred in males, while a significant increase of nociceptive temporal summation following sleep deprivation mostly occurred in females. Furthermore, Eichhorn et al. (2018) observed that the decrease in endogenous inhibitory control associated with sleep deprivation only occurred in females. From those results as well as ours, it is clear that there are not only significant differences in nociception and pain between the sexes (Bartley and Fillingim 2013), but also that the effect of sleep deprivation on nociceptive processing and pain might depend on sex.

### Limitations

There are several limitations that should be addressed before adopting this method in further clinical or pharmacological studies. This was an exploratory study, as this was the first study to examine intra-epidermal electric detection thresholds and EPs following sleep deprivation, and no prior data were available to formulate hypotheses and perform a sample size calculation. Although this study included a larger group of participants than earlier studies showing significant effects of sleep deprivation on nociceptive detection thresholds [ranging from 6 (Moldofsky and Scarisbrick 1976) to 20 (Kundermann et al. 2004) participants] or EPs [ranging from 12 (Schuh-Hofer et al. 2015) to 33 (Ødegård et al. 2015) participants], this study might still lack sufficient power to observe some of the sex-dependent effects of sleep deprivation.

Several other choices in our current study design might have impacted study results, and are important to address in potential follow-up studies. In the current study, the male and female population were recruited in two time periods with an interval of 1.5 years. As such, potential confounding by the time period in which the experiments were performed (e.g., COVID-19 risk mitigation measures, seasonal effects, and potentially other unknown factors) on the sex-dependent effects observed in this study, cannot be excluded. Follow-up studies should therefore recruit and test participants in the same time period. Females were required to use their own hormonal contraception continuously during study participation to prevent an influence of potential hormonal variations caused by the menstrual cycle on pain perception (Kowalczyk et al. 2010). Nevertheless, this might limit generalizability of our current observations to females who do not take hormonal contraception. The effect of hormones on nociceptive processing following sleep deprivation remains undocumented, and further studies are needed to provide more insight in the potential influence of hormones on sleep and nociception. Another potential bias in outcomes might have been introduced by the time gap between occasions. As in half of the subjects, the second occasion was preceded by a resting period of at least 5 days, while in the other half, the second occasion was preceded by the first (separated by one night), this could have led to a bias in outcomes due to potential familiarization effects in the second half. Future experiments might avoid such a bias by including an equal resting period between each occasion. Experiments with male and female participants were performed by a mixed population of research assistants of both sexes. As the gender of the experimenter can influence reported pain measures (Aslaksen et al. 2007; Kállai et al. 2004; Levine and Lee De Simone 1991), this could have led to additional variance of outcomes between subjects.

## Conclusion

Observation of altered nociceptive detection thresholds and EPs following sleep deprivation in male and female populations shows that it is feasible to evaluate impaired nociceptive processing following sleep deprivation in a human population based on intra-epidermal detection thresholds and EPs. Some effects were only observed in either a male or a female population, such as a decrease of the intra-epidermal electric detection threshold or a decrease of the EP, and might be sex-dependent. The current results suggest that intra-epidermal electric detection thresholds and EPs could be helpful in exploring the link between sleep impairment and chronic pain in future studies. Nevertheless, it remains important to note that, like any method relying on participant report (e.g., questionnaires, quantitative sensory testing), nociceptive detection thresholds and EPs might be influenced by attention and learning processes. Developing nociception biomarkers that are unbiased by psychological states remains a current challenge for pain science. The possibilities of combining the sleep deprivation model with more objective measures of nociception and pain are exciting, as they allow to translate results from earlier pharmacological animal studies using sleep deprivation, e.g., (Gürel et al. 2014; Skinner et al. 2011; Wodarski et al. 2015), to humans with potential applications in the identification of analgesic and sedative compounds.

## Data Availability

The data that support the findings of this study are available on request from the corresponding author. The data are not publicly available due to privacy and ethical restrictions.

## Appendix I

Covariance matrices of (generalized) linear mixed models.

See Tables 5, 6, 7, 8, 9 and 10.

**Table 5:** Random-effects covariance matrix of the generalized linear mixed model fit (Table 1) for the combination of the male and the female group

	(Intercept)	Amp	Sleep dep	Type 2	Trial num	Measurement 2	Occasion 2	Amp. × Sleep dep	Amp. × Type 2	Type 2 × Sleep dep	Trial num. × Sleep dep	Amp. × Type 2 × Sleep dep
(Intercept)	0.071	− 0.043	− 0.084	− 0.015	0.000	− 0.041	− 0.021	0.143	0.078	0.037	− 0.010	− 0.125
Amp	− 0.043	0.251	0.057	0.016	− 0.012	0.018	-0.028	− 0.210	0.148	− 0.046	0.012	0.029
Sleep dep	− 0.084	0.057	0.179	0.015	− 0.002	0.066	0.006	− 0.389	− 0.082	− 0.050	0.016	− 0.009
Type 2	− 0.015	0.016	0.015	0.025	0.001	0.007	0.002	− 0.023	− 0.100	− 0.038	0.002	0.080
Trial num	0.000	− 0.012	− 0.002	0.001	0.003	0.001	0.004	0.005	− 0.022	0.004	− 0.001	− 0.005
Measurement 2	− 0.041	0.018	0.066	0.007	0.001	0.070	− 0.009	− 0.076	− 0.035	− 0.008	0.004	0.013
Occasion 2	-0.021	− 0.028	0.006	0.002	0.004	− 0.009	0.041	0.016	− 0.063	− 0.011	− 0.001	0.134
Amp. × Sleep dep	0.143	− 0.210	− 0.389	− 0.023	0.005	-0.076	0.016	0.398	0.098	0.107	− 0.038	0.694
Amp. × Type 2	0.078	0.148	− 0.082	− 0.100	− 0.022	− 0.035	− 0.063	0.098	0.847	0.141	− 0.011	− 0.402
Type 2 × Sleep dep	0.037	− 0.046	− 0.050	− 0.038	0.004	− 0.008	− 0.011	0.107	0.141	0.164	0.000	− 0.419
Trial num. × Sleep dep	− 0.010	0.012	0.016	0.002	− 0.001	0.004	− 0.001	− 0.038	− 0.011	0.000	0.006	− 0.009
Amp. × Type 2 × Sleep dep	− 0.125	0.029	− 0.009	0.080	− 0.005	0.013	0.134	0.694	− 0.402	− 0.419	− 0.009	2.711

**Table 6:** Random-effects covariance matrix of the generalized linear mixed model fit (Table 1) for the female group

	(Intercept)	Amp	Sleep dep	Type 2	Trial num	Measurement 2	Occasion 2	Amp. × Sleep dep	Amp. × Type 2	Type 2 × Sleep dep	Trial num. × Sleep dep	Amp. × Type 2 × Sleep dep
(Intercept)	0.171	− 0.178	− 0.213	− 0.042	0.011	− 0.124	− 0.004	0.340	0.107	0.102	− 0.029	− 0.221
Amp	− 0.178	0.577	0.222	0.069	− 0.022	0.060	− 0.027	− 0.684	0.077	− 0.191	0.031	0.216
Sleep dep	− 0.213	0.222	0.364	0.057	− 0.018	0.148	− 0.026	− 0.748	− 0.179	− 0.155	0.046	0.133
Type 2	− 0.042	0.069	0.057	0.052	− 0.001	0.023	− 0.005	− 0.126	− 0.225	− 0.099	0.009	0.142
Trial num	0.011	− 0.022	− 0.018	− 0.001	0.004	− 0.001	0.001	0.041	− 0.012	0.018	− 0.003	− 0.028
Measurement 2	− 0.124	0.060	0.148	0.023	− 0.001	0.139	− 0.009	− 0.134	− 0.094	− 0.011	0.016	0.044
Occasion 2	− 0.004	− 0.027	− 0.026	− 0.005	0.001	− 0.009	0.047	0.054	0.006	− 0.004	0.003	0.097
Amp. × Sleep dep	0.340	− 0.684	− 0.748	− 0.126	0.041	− 0.134	0.054	3.326	0.189	0.520	− 0.093	0.566
Amp. × Type 2	0.107	0.077	− 0.179	− 0.225	− 0.012	− 0.094	0.006	0.189	1.601	0.407	− 0.037	− 0.826
Type 2 × Sleep dep	0.102	− 0.191	− 0.155	− 0.099	0.018	− 0.011	− 0.004	0.520	0.407	0.509	− 0.020	− 1.296
Trial num. × Sleep dep	− 0.029	0.031	0.046	0.009	− 0.003	0.016	0.003	− 0.093	− 0.037	− 0.020	0.012	0.026
Amp. × Type 2 × Sleep dep	− 0.221	0.216	0.133	0.142	− 0.028	0.044	0.097	0.566	− 0.826	− 1.296	0.026	6.909

**Table 7:** Random-effects covariance matrix of the generalized linear mixed model fit (Table 1) for the male group

	(Intercept)	Amp	Sleep dep	Type 2	Trial num	Measurement 2	Occasion 2	Amp. × Sleep dep	Amp. × Type 2	Type 2 × Sleep dep	Trial num. × Sleep dep	Amp. × Type 2 × Sleep dep
(Intercept)	0.127	− 0.034	− 0.073	− 0.027	− 0.008	0.014	− 0.086	0.045	0.235	0.018	0.004	− 0.156
Amp	− 0.034	0.232	0.092	− 0.020	− 0.016	0.002	− 0.080	− 0.082	0.325	0.032	0.028	− 0.063
Sleep dep	− 0.073	0.092	0.440	− 0.006	0.001	0.104	− 0.118	− 0.556	0.095	− 0.066	− 0.004	− 0.139
Type 2	− 0.027	− 0.020	− 0.006	0.061	0.003	0.009	0.035	0.108	− 0.184	− 0.080	− 0.012	0.298
Trial num	− 0.008	− 0.016	0.001	0.003	0.007	0.000	0.013	0.003	− 0.060	0.000	− 0.004	0.020
Measurement 2	0.014	0.002	0.104	0.009	0.000	0.115	− 0.053	− 0.150	0.058	− 0.049	− 0.006	− 0.043
Occasion 2	− 0.086	− 0.080	− 0.118	0.035	0.013	− 0.053	0.235	0.137	− 0.330	− 0.034	− 0.022	0.280
Amp. x Sleep dep	0.045	− 0.082	− 0.556	0.108	0.003	− 0.150	0.137	1.591	− 0.204	− 0.067	− 0.040	1.186
Amp. x Type 2	0.235	0.325	0.095	− 0.184	− 0.060	0.058	− 0.330	− 0.204	1.592	0.153	0.049	− 1.003
Type 2 × Sleep dep	0.018	0.032	− 0.066	− 0.080	0.000	− 0.049	− 0.034	− 0.067	0.153	0.210	0.027	− 0.617
Trial num. x Sleep dep	0.004	0.028	− 0.004	− 0.012	− 0.004	− 0.006	− 0.022	− 0.040	0.049	0.027	0.018	− 0.083
Amp. x Type 2 × Sleep dep	− 0.156	− 0.063	− 0.139	0.298	0.020	− 0.043	0.280	1.186	− 1.003	− 0.617	− 0.083	3.571

**Table 8:** Random-effects covariance matrix of the linear mixed model fit (Table 3) for the combination of the male and the female group

	(Intercept)	Amp	Amp. × Sleep dep	Amp. × Type 2	Amp. × Type 2 × Sleep dep	Measurement 2	Occasion 2	Detected	Detected × Sleep dep	Detected × Sleep dep. x Trial num	Detected × Trial num	Sleep dep	Sleep dep. × Trial num	Trial num	Type 2	Type 2 × Sleep dep
(Intercept)	5.608	− 0.171	0.841	0.319	− 0.422	− 0.455	− 0.330	0.060	− 0.046	− 0.561	0.805	− 0.843	0.575	− 0.790	− 0.436	0.644
Amp	0.171	3.109	− 0.203	0.659	− 0.663	− 0.230	0.459	0.539	-0.321	0.669	− 0.300	0.014	− 0.674	0.251	− 0.090	0.178
Amp. x Sleep dep	0.841	− 0.203	8.642	0.357	− 0.376	− 0.338	− 0.166	− 0.076	0.136	− 0.305	0.606	− 0.941	0.429	− 0.529	− 0.454	0.788
Amp. x Type 2	0.319	0.659	0.357	7.024	− 0.856	− 0.341	0.619	0.317	− 0.035	0.551	− 0.083	− 0.414	− 0.363	− 0.047	− 0.370	0.397
Amp. x Type 2 × Sleep dep	− 0.422	− 0.663	− 0.376	− 0.856	11.252	0.621	− 0.451	− 0.511	0.171	− 0.268	− 0.174	0.557	0.187	0.115	0.451	− 0.570
Measurement 2	− 0.455	− 0.230	− 0.338	− 0.341	0.621	4.821	− 0.260	− 0.141	0.179	0.192	− 0.348	0.603	0.090	0.068	0.228	− 0.524
Occasion 2	− 0.330	0.459	− 0.166	0.619	− 0.451	− 0.260	2.736	0.114	0.266	0.708	− 0.592	0.108	− 0.761	0.507	0.206	− 0.179
Detected	0.060	0.539	− 0.076	0.317	− 0.511	− 0.141	0.114	8.231	− 0.440	0.033	− 0.160	− 0.027	− 0.172	0.087	0.110	0.030
Detected x Sleep dep	− 0.046	− 0.321	0.136	− 0.035	0.171	0.179	0.266	− 0.440	5.490	0.050	0.067	− 0.011	− 0.041	− 0.244	0.387	− 0.213
Detected x Sleep dep. x Trial num	− 0.561	0.669	− 0.305	0.551	− 0.268	0.192	0.708	0.033	0.050	5.077	− 0.717	0.294	− 0.781	0.602	− 0.018	− 0.096
Detected x Trial num	0.805	− 0.300	0.606	− 0.083	− 0.174	− 0.348	− 0.592	− 0.160	0.067	− 0.717	0.011	− 0.652	0.708	− 0.864	− 0.383	0.541
Sleep dep	− 0.843	0.014	− 0.941	− 0.414	0.557	0.603	0.108	− 0.027	− 0.011	0.294	− 0.652	9.577	− 0.331	0.474	0.506	− 0.896
Sleep dep. x Trial num	0.575	− 0.674	0.429	− 0.363	0.187	0.090	− 0.761	− 0.172	− 0.041	− 0.781	0.708	− 0.331	0.563	− 0.618	− 0.452	0.277
Trial num	− 0.790	0.251	− 0.529	− 0.047	0.115	0.068	0.507	0.087	− 0.244	0.602	− 0.864	0.474	− 0.618	1.491	0.163	− 0.268
Type 2	− 0.436	− 0.090	− 0.454	− 0.370	0.451	0.228	0.206	0.110	0.387	− 0.018	− 0.383	0.506	− 0.452	0.163	1.665	− 0.727
Type 2 × Sleep dep	0.644	0.178	0.788	0.397	− 0.570	− 0.524	− 0.179	0.030	− 0.213	− 0.096	0.541	− 0.896	0.277	− 0.268	− 0.727	5.818

**Table 9:** Random-effects covariance matrix of the linear mixed model fit (Table 3) for the female group

	(Intercept)	Amp	Amp. x Sleep dep	Amp. x Type 2	Amp. x Type 2 × Sleep dep	Measurement 2	Occasion 2	Detected	Detected x Sleep dep	Detected x Sleep dep. x Trial num	Detected x Trial num	Sleep dep	Sleep dep. x Trial num	Trial num	Type 2	Type 2 × Sleep dep
(Intercept)	2.025	− 0.670	0.765	0.099	− 0.089	− 0.313	0.335	0.527	− 0.095	− 0.274	00.342	− 0.522	0.106	− 0.504	− 0.344	0.462
Amp	− 0.670	2.593	− 0.665	0.148	0.374	− 0.303	− 0.139	− 0.794	0.032	− 0.022	0.039	0.279	0.191	0.146	− 0.025	− 0.354
Amp. x Sleep dep	0.765	− 0.665	9.205	0.251	− 0.400	− 0.402	0.590	0.741	− 0.069	0.217	− 0.133	− 0.832	− 0.433	0.038	− 0.293	0.604
Amp. x Type 2	0.099	0.148	0.251	10.780	− 0.559	− 0.621	0.675	0.139	0.254	0.330	− 0.329	− 0.282	− 0.312	0.218	− 0.630	0.132
Amp. x Type 2 × Sleep dep	− 0.089	0.374	− 0.400	− 0.559	19.056	00.111	− 0.568	− 0.605	0.236	− 0.636	00.584	0.364	0.434	− 0.605	0.574	− 0.584
Measurement 2	− 0.313	− 0.303	− 0.402	− 0.621	0.111	30.995	− 0.719	0.008	0.041	− 0.091	-0.110	0.656	0.149	0.028	0.499	− 0.241
Occasion 2	0.335	− 0.139	0.590	0.675	− 0.568	-0.719	3.519	0.260	0.170	0.565	− 0.136	− 0.793	− 0.573	0.166	− 0.512	0.405
Detected	0.527	− 0.794	0.741	0.139	− 0.605	0.008	0.260	0.927	− 0.246	0.200	− 0.443	− 0.384	− 0.365	0.288	− 0.094	0.435
Detected x Sleep dep	− 0.095	0.032	− 0.069	0.254	0.236	0.041	0.170	− 0.246	5.857	0.158	− 0.052	0.145	− 0.444	− 0.244	0.374	− 0.620
Detected x Sleep dep. x Trial num	− 0.274	− 0.022	0.217	0.330	− 0.636	-0.091	0.565	0.200	0.158	5.526	− 0.729	− 0.413	− 0.844	0.770	− 0.163	0.328
Detected x Trial num	0.342	0.039	− 0.133	− 0.329	0.584	-0.110	− 0.136	− 0.443	− 0.052	− 0.729	2.993	0.035	0.691	− 0.890	− 0.005	− 0.130
Sleep dep	− 0.522	0.279	− 0.832	− 0.282	0.364	00.656	− 0.793	− 0.384	0.145	− 0.413	0.035	5.838	0.471	− 0.148	0.415	− 0.671
Sleep dep. x Trial num	0.106	0.191	− 0.433	− 0.312	0.434	00.149	− 0.573	− 0.365	− 0.444	− 0.844	0.691	00.471	2.928	− 0.587	− 0.158	− 0.063
Trial num	− 0.504	0.146	0.038	0.218	− 0.605	0.028	0.166	0.288	− 0.244	− 0.770	− 0.890	− 0.148	− 0.587	1.642	− 0.098	0.296
Type 2	− 0.344	− 0.025	− 0.293	− 0.630	0.574	00.499	− 0.512	− 0.094	0.374	− 0.163	− 0.005	00.415	− 0.158	− 0.098	2.733	− 0.720
Type 2 × Sleep dep	0.462	− 0.354	0.604	0.132	− 0.584	-0.241	0.405	0.435	− 0.620	0.328	− 0.130	− 0.671	− 0.063	0.296	− 0.720	5.888

**Table 10:** Random-effects covariance matrix of the linear mixed model fit (Table 3) for the male group

	(Intercept)	Amp	Amp. x Sleep dep	Amp. x Type 2	Amp. x Type 2 × Sleep dep	Measurement 2	Occasion 2	Detected	Detected x Sleep dep	Detected x Sleep dep. x Trial num	Detected x Trial num	Sleep dep	Sleep dep. x Trial num	Trial num	Type 2	Type 2 × Sleep dep
(Intercept)	8.639	− 0.442	0.936	0.256	0.392	− 0.476	− 0.303	− 0.250	0.512	− 0.531	0.787	− 0.939	0.528	− 0.832	− 0.251	0.442
Amp	− 0.442	6.528	− 0.608	0.433	− 0.548	− 0.184	− 0.029	0.732	− 0.592	0.337	− 0.116	0.381	− 0.295	0.035	0.775	− 0.140
Amp. x Sleep dep	0.936	− 0.608	12.474	0.199	0.528	− 0.369	− 0.130	− 0.384	0.649	− 0.381	0.586	− 0.935	0.489	− 0.605	− 0.327	0.492
Amp. x Type 2	0.256	0.433	0.199	3.420	0.162	− 0.329	0.454	0.219	− 0.217	0.434	− 0.020	− 0.223	− 0.434	− 0.219	0.746	− 0.146
Amp. x Type 2 × Sleep dep	0.392	− 0.548	0.528	0.162	6.468	0.515	0.198	− 0.277	0.274	0.159	− 0.128	− 0.231	0.227	− 0.083	− 0.115	− 0.231
Measurement 2	− 0.476	− 0.184	− 0.369	− 0.329	0.515	5.186	0.082	− 0.132	− 0.038	0.431	− 0.584	0.615	− 0.015	0.463	− 0.162	− 0.490
Occasion 2	− 0.303	− 0.029	− 0.130	0.454	0.198	0.082	2.800	− 0.076	− 0.363	0.559	− 0.739	0.298	− 0.828	0.645	0.450	− 0.540
Detected	-0.250	0.732	− 0.384	0.219	− 0.277	− 0.132	− 0.076	9.650	− 0.651	− 0.089	− 0.030	0.204	0.052	− 0.028	0.742	− 0.278
Detected x Sleep dep	0.512	− 0.592	0.649	− 0.217	0.274	− 0.038	− 0.363	− 0.651	4.750	− 0.104	0.412	− 0.617	0.508	− 0.309	− 0.693	0.772
Detected x Sleep dep. x Trial num	− 0.531	0.337	− 0.381	0.434	0.159	0.431	0.559	− 0.089	− 0.104	4.784	− 0.736	0.495	− 0.610	0.580	0.409	− 0.235
Detected x Trial num	0.787	− 0.116	0.586	− 0.020	− 0.128	− 0.584	− 0.739	− 0.030	0.412	− 0.736	3.131	− 0.767	0.671	− 0.939	− 0.313	0.595
Sleep dep	− 0.939	0.381	− 0.935	− 0.223	− 0.231	0.615	0.298	0.204	− 0.617	0.495	− 0.767	11.826	− 0.543	0.734	0.240	− 0.662
Sleep dep. x Trial num	0.528	− 0.295	0.489	− 0.434	0.227	− 0.015	− 0.828	0.052	0.508	− 0.610	0.671	− 0.543	2.925	− 0.600	− 0.480	0.543
Trial num	− 0.832	0.035	− 0.605	− 0.219	− 0.083	0.463	0.645	− 0.028	− 0.309	0.580	− 0.939	0.734	− 0.600	1.401	0.127	− 0.412
Type 2	− 0.251	0.775	− 0.327	0.746	− 0.115	− 0.162	0.450	0.742	− 0.693	0.409	− 0.313	0.240	− 0.480	0.127	1.969	− 0.440
Type 2 × Sleep dep	0.442	− 0.140	0.492	− 0.146	− 0.231	− 0.490	− 0.540	− 0.278	0.772	− 0.235	0.595	− 0.662	0.543	− 0.412	− 0.440	4.816

## Code availability

Code required to perform the analyses reported here is available on request.
